# Mmu-miR-615-3p Regulates Lipoapoptosis by Inhibiting C/EBP Homologous Protein

**DOI:** 10.1371/journal.pone.0109637

**Published:** 2014-10-14

**Authors:** Yasuhiro Miyamoto, Amy S. Mauer, Swarup Kumar, Justin L. Mott, Harmeet Malhi

**Affiliations:** 1 Division of Gastroenterology and Hepatology, Mayo Clinic, Rochester, Minnesota, United States of America; 2 Department of Biochemistry and Molecular Biology, University of Nebraska Medical Center, Omaha, Nebraska, United States of America; University of Hong Kong, Hong Kong

## Abstract

Lipoapoptosis occurring due to an excess of saturated free fatty acids such as palmitate is a key pathogenic event in the initiation of nonalcoholic fatty liver disease. Palmitate loading of cells activates the endoplasmic reticulum stress response, including induction of the proapoptotic transcription factor C/EBP homologous protein (CHOP). Furthermore, the loss of microRNAs is implicated in regulating apoptosis under conditions of endoplasmic reticulum (ER) stress. The aim of this study was to identify specific microRNAs regulating CHOP expression during palmitate-induced ER stress. Five microRNAs were repressed under palmitate-induced endoplasmic reticulum stress conditions in hepatocyte cell lines (miR-92b-3p, miR-328-3p, miR-484, miR-574-5p, and miR-615-3p). We identified miR-615-3p as a candidate microRNA which was repressed by palmitate treatment and regulated CHOP protein expression, by RNA sequencing and *in silico* analyses, respectively. There is a single miR-615-3p binding site in the 3′untranslated region (UTR) of the *Chop* transcript. We characterized this as a functional binding site using a reporter gene-based assay. Augmentation of miR-615-3p levels, using a precursor molecule, repressed CHOP expression; and under these conditions palmitate- or tunicamycin-induced cell death were significantly reduced. Our results suggest that palmitate-induced apoptosis requires maximal expression of CHOP which is achieved via the downregulation of its repressive microRNA, miR-615-3p. We speculate that enhancement of miR-615-3p levels may be of therapeutic benefit by inhibiting palmitate-induced hepatocyte lipoapoptosis.

## Introduction

The molecular pathogenesis of the highly prevalent chronic liver disease, nonalcoholic fatty liver disease (NAFLD) is not fully understood [1], [2]. Progressive forms of NAFLD, termed nonalcoholic steatohepatitis (NASH) are characterized by hepatocyte apoptosis, which correlates with disease severity as well as disease progression to cirrhosis [3]. Circulating free fatty acids (FFA) are elevated in NASH, and when elevated induce apoptosis of cells, a process termed lipoapoptosis [4], [5]. It is postulated that FFA-induced hepatocyte apoptosis is a key pathogenic event in the progression of NASH, which is increasingly viewed as a lipotoxic disease. Recent studies have linked endoplasmic reticulum (ER) stress and microRNAs (miRs) to NAFLD.

MicroRNAs are small noncoding RNAs increasingly recognized in modulating the cellular response to stress [6]. MicroRNAs bind to complementary seed sequences in the 3′untranslated region (3′UTR) of their target mRNA, resulting in either target mRNA degradation, or attenuation of translation. Thus, by post-transcriptionally regulating the expression of their target proteins, microRNAs are able to fine tune cellular protein levels and thus a cell's response to stress. MicroRNA profiling has shown that microRNAs are modified in NAFLD in humans and in rodent models, however, the functional consequences of these changes have not been fully elucidated [7], [8]. One of the described links between microRNAs and lipoapoptosis, demonstrated that microRNA-296 contributed to apoptosis by targeting the proapoptotic protein PUMA [9]. Furthermore, recent studies have linked microRNAs to ER stress pathways; however, the role of microRNAs in regulating ER stress-induced cell death under lipotoxic conditions has not been explored.

*ER stress is a recognized feature of NAFLD, and one of the molecular pathways implicated in hepatocyte lipoapoptosis downstream of ER stress is C/EBP homologous protein (CHOP)-mediated cell death [10]. In addition, palmitate can activate the ER stress response, including CHOP expression in many cell types, including Chinese hamster ovary cells, cardiomyocytes, fibroblasts, INS-1E cell line and pancreatic beta cells [11], [12], [13]. In these models increased ER membrane saturated lipid content and ER calcium depletion are implicated as mechanisms. CHOP is a transcription factor, expressed at very low levels under normal conditions [14]. Cellular stress, including endoplasmic reticulum (ER) stress transcriptionally activates the expression of CHOP, wherein, CHOP is implicated in the ensuing apoptosis [15]. Furthermore, mice deficient in CHOP (Chop-/-) are protected from tunicamycin-induced liver and kidney injury. Increased protein synthesis rates and modulation of the death receptor, death receptor 5, and Bcl-2 family proteins have been proposed as possible mechanisms for CHOP-dependent cell death [16], [17], [18], [19]. Furthermore, recent studies have linked microRNAs to the regulation of ER stress-induced cell death [20]. However, the specific role of microRNAs that repress CHOP under lipotoxic conditions remains unexplored.*

In this study we have identified that the lipotoxic saturated free fatty acid, palmitate, induces ER stress, and a concomitant decrease in microRNA miR-615-3p levels, thus derepressing CHOP protein expression. Using gain-of-function and loss-of-function approaches we demonstrate that this microRNA regulates CHOP expression and cell death. Analysis of mouse liver samples with NASH demonstrates decreased miR-61-3p levels with concomitant increase in CHOP protein levels.

## Materials and Methods

### Cells

Neonatal mouse hepatocytes were derived from wild-type or IRE1α knockout mice, as previously described, and immortalized with SV40 large T antigen and were a gift from Dr. Randal Kaufman [21]. The mouse hepatoma cell line Hepa 1–6 was obtained from ATCC. Primary mouse hepatocytes were isolated and percoll purified from C57BL/6 male mice by collagenase-based liver perfusion as previously described, and utilized for experiments only if greater than 95% viable [22]. The use of mice for isolation of hepatocytes was reviewed and approved by the Institutional Animal Use and Care Committee, Mayo Clinic, Rochester. The well-established human liver cell line, Huh7 (JCRB Cell Bank, # JCRB0403, derived from a well-differentiated hepatocellular carcinoma from a 57 year old male) was cultured as described [23].

### Fatty acid treatment

Palmitic acid (PA) was purchased from Sigma-Aldrich. Albumin-bound PA was applied to cells as previously described by us [24].

### MicroRNA expression analysis

MicroRNAs were isolated from cells using the miRNeasy mini kit (Qiagen) according to the manufacturer's instructions. Sequencing was performed at the Medical Genomics Facility, Mayo Clinic, Rochester, MN. Briefly, microRNAs were sequenced using the NEBNext Small RNA library prep kit on an Illumina HiSeq 2000. The Illumina reads were trimmed of adapters with Cutadapt [25]). Trimmed microRNA sequences greater than 17 nucleotides in length were then aligned to the mm9 reference genome and the miRBase v19 reference sequences using Bowtie [26]. Known microRNA expression and novel microRNA prediction were quantified using the miRDeep2 package [27]. Differential expression analysis was performed with the Bioconductor R package EdgeR [28]. Individual microRNAs were amplified by Taqman assays (Applied Biosystems) for mmu-miR-615-3p and has-miR-615-3p (P131015-004E01) and normalized to the expression of sno-202 (P131022-004H07) for mouse, and U47 (P140221-004G06) for human, to calculate the dCT. Relative expression was expressed as fold change using the ddCT method, as described [29].

### Total RNA isolation and quantitative PCR

Total RNA was isolated from cells using the RNeasy mini kit (Qiagen) according to the manufacturer's instructions. Reverse transcription was performed using the iScript cDNA synthesis kit (BioRad). Quantitative real-time PCR (qPCR) reactions were run on the LightCycler 480 (Roche), using the LightCycler 480 SYBR Green 1 Master (Roche). The following primers were used: Chop FP-5′-CTGCCTTTCACCTTGGAGAC-3′ and RP-5′-CGTTTCCTGGGGATGAGATA-3′, and HPRT FP- 5′-TCCTCCTCAGACCGCTTTT-3′ and RP 5′-CCTGGTTCATCATCGCTAATC-3′. Relative mRNA expression was expressed using the method of Livak and Schmittgen [29].

### Transfection and Labeling

Cultured cells were transfected with Opti-MEM I Reduced-Serum Medium (catalog#31985-070, Life Tecnologies, Grand Island, NY), X-treme Gene siRNA Transfection Reagent(catalog#0447609300, Roche, Mannheim, Germany), miRCURY LNA Inhibitor Control (Negative control A catalog#199004-00, 5nmol, EXIQON, Woburn, MA), miRCURY LNA Inhibitor (hsa-miR-615-3p catalog#411356-00, Batch#224134, 5 nmol, EXIQON, Woburn, MA), mirVana microRNA mimic negative control #1(catalog#4464058, Lot#ASO0VDIQ, 5 nmol, Ambion, Grand Island, NY), mirVana microRNA mimic hsa-miR-615 (catalog#4464066, 5 nmol, Ambion, Grand Island, NY). Using Ambion by Life Technologies Silencer siRNA Labeling Kit-Cy3 (cat # AM1632) according to manufacturer's instructions for labeling duplex siRNA we labeled 50 µM negative control #1 or mirVana microRNA mir-615-3p mimic.

### Protein isolation and Western Blotting

Proteins were extracted using RIPA buffer, resolved by SDS PAGE on commercially available Criterion acrylamide gels (BioRad), and electroblotted onto polyvinylidene fluoride (PVDF) membrane. 5% non-fat dairy milk in Tris-buffered saline (20 mM Tris, 150 mM NaCl, pH 7.4) with 0.1% Tween-20 or LICOR Blocking Buffer (LiCor Biosciences, Lincoln, NE) were used to block non-specific binding sites. Blots were incubated with primary antibodies at 4°C overnight and with secondary antibodies at ambient temperature for 1 hour. The following primary antibodies were used: mouse monoclonal Ab to CHOP (sc-7315, 1∶100, Santa Cruz Biotechnology, Dallas, TX), rabbit polyclonal Ab to CHOP (sc-793, 1∶100, Santa Cruz), rabbit polyclonal Ab to activating transcription factor-4 (ATF4) (sc-200, 1∶200, Santa Cruz), rabbit polyclonal Ab to the alpha subunit of eukaryotic initiation factor phosphorylated at serine 52 (p-eIF2α, 44–728G, 1∶1000, Invitrogen), rabbit polyclonal antibody to eIF2α (9722, 1∶1000, Cell Signaling), mouse monoclonal Ab to glyceraldehyde-3-phosphate dehydrogenase (GAPDH, MAB374, 1∶10,000, Millipore) and γ-Tubulin (T6557, 1∶5000, Sigma Pharmaceuticals, St. Louis, MO). HRP conjugated secondary antibodies; goat anti-mouse (sc-2005, 1∶3000, Santa Cruz) and goat anti-rabbit (sc-2004, 1∶3000, Santa Cruz) were used to detect antigen-antibody complexes. Infrared dye conjugated secondary antibodies; donkey anti-rabbit (926–32213, 1∶20,000, LiCor) and donkey anti mouse (926–68072, 1∶20,000, LiCor) were used for detection by the Odyssey infrared imager. Immune complexes were visualized using a chemilunimescent substrate (ECL, Amersham, IL), and Kodak XOMAT film (Eastman Kodak, Rochester, NY) or by Odyssey Imager (LI COR Biosciences, Lincoln, Nebraska). NIH Image J software was used for densitometric quantification of CHOP expression, consistently under induced conditions, and when detected under basal conditions.

### Luciferase Assay

The region of the wild-type *Chop* mRNA (also known as *Ddit3*) 3′UTR with a putative miR-615-3p binding site or mutant *Chop* mRNA 3′UTR were cloned into pMIR-REPORT Luciferase vector (cat # AM5795, Applied Biosystems) using *Spe I* and *Hind III* sites. The sequences of the putative binding site and the regions targeted by mutagenesis and cloned into the reporter gene are depicted in Figure S1. All plasmids were verified by sequencing. These constructs were transfected into Hek293A cells using Lipofectamine LTX with Plus Reagent (cat #18324-012, Life Technologies). Cells were plated at a density of 3600/cm^2^ {(1×10^4^) per well} per well, into a 96-well plate and attached overnight. They were co-transfected with 100 ng of wild-type or mutant reporter vector, 10 ng of internal control pRL-TK-Renilla-luciferase plasmid (cat# E2241, Promega) and negative control #1 or mirvana microRNA miR-615-3p mimic, both from Life Technologies final concentration, 80 nM. Twenty four hours post-transfection, luciferase activities were measured using the Dual-Glo Luciferase Assay System (cat # E2920, Promega) according to the manufacturer's instructions. Firefly luciferase values were normalized by dividing by the *Renilla* luciferase values.

### Apoptosis assessment

Apoptosis was quantified by nuclear morphologic changes of apoptosis following staining with 4′,6-diamidino-2-phenylindole (DAPI). Apoptotic cells were counted under fluorescence microscopy, and expressed as a percent of total cells. Three hundred to four hundred total cells were counted per condition per replicate. For apoptosis assessment with the mirVana miR-615-3p mimic, only cells which were transfected with the fluorescently labeled mimic were included. Six hours after the transfection, cells were treated with either palmitate or tunicamycin for 18 hours, fixed using 10% neutral buffered formaldehyde, mounted in Prolong Antifade Gold with DAPI (cat # P36935, Life Technologies) and counted under fluorescence microscopy. To confirm apoptosis, caspase 3/7 activity was measured biochemically using a commercially available homogeneous luminescent assay, Caspase 3/7-Glo (cat # G-8091, Promega Corporation, Madison, WI). Briefly, an equal volume of the reaction mixture (containing lysis buffer and a proluminescent caspase 3/7 substrate) was added to each well of the 96 well plate, and luminescence was acquired with a plate reader (Biotek Corporation,

### Mouse Liver Studies

Archived total RNA from mice fed either chow (n = 7) or the high fructose, high fat, high cholesterol diet (n = 13), and whole liver protein extracts from chow-fed mice (n = 3) and high fructose, high fat, high cholesterol diet-fed mice (n = 8) were a kind gift of Dr. Charlton. This is a bona fide model of human NASH, recapitulating key metabolic and liver features of this disease [30]. The studies conducted by Dr. Charlton et al were done with review and approval by the Institutional Animal Care and Use Committee, Mayo Clinic.

### Reagents

4′, 6-diamidino-2-phenylindole (DAPI), isopropanol, bovine serum albumin, and palmitic acid were from Sigma Pharmaceuticals (St. Louis, MO). Tunicamycin was from Calbiochem.

### Statistical Analysis

Data are presented as the means ±S.E.M. from three or more independent experiments unless indicated otherwise. Statistical analysis was performed using two-tailed t tests. p<0.05 was considered a significant difference.

## Results

### MicroRNAs decreased by palmitate treatment

*Due to our interest in lipotoxicity and endoplasmic reticulum (ER) stress, we utilized previously described mouse hepatocyte-derived cell lines to identify microRNAs (miRs) that were downregulated by palmitate treatment and tunicamycin-induced ER stress [21]. The cell lines utilized were derived from wild-type hepatocytes (IRE-WT) and from hepatocytes deficient in IRE1α (IRE-KO) expression. We first identified microRNAs downregulated by palmitate (400 µM, 16 hours) or tunicamycin (1 µg/mL, 16 hours) in IRE-WT and IRE-KO hepatocytes by pair-wise comparisons of microRNAs downregulated under these conditions, compared to vehicle treated cells (Data S1-S4). We employed this approach in order to avoid identifying microRNAs that were potentially targeted by the recently described microRNA-processing activity of IRE1α [20]. We identified five common miRs which were downregulated by both palmitate and tunicamycin in both wild-type and IRE1α deficient hepatocytes (Table 1).*

**Table 1:** MicroRNAs downregulated by palmitate and tunicamycin treatment in IRE1α wild-type and knockout cells.

MicroRNA
Mmu-miR-92b-3p
Mmu-miR-574-5p
Mmu-miR-615-3p
Mmu-miR-484
Mmu-miR-328-3p

**Figure 1:**
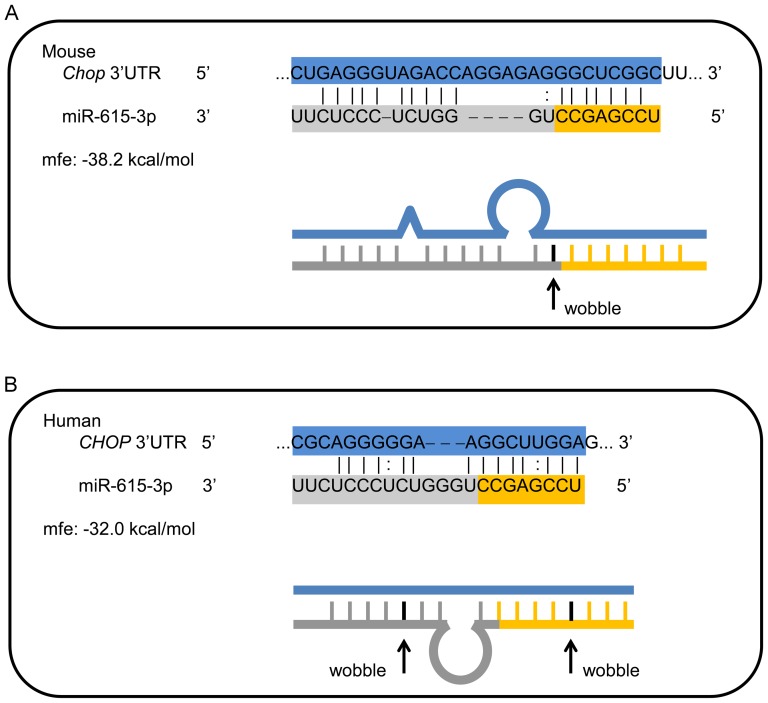
Computationally identified miR-615-3p binding site in *Chop* mRNA. (A) Schematic representation of the sequence for mouse *Chop* mRNA showing a section of the 3′ untranslated region (UTR) and the mmu-miR-615-3p sequence below. The 7-mer binding site is depicted by the solid lines at the 3′ end of the *Chop* mRNA. (B) Sequence of a section of the human 3′UTR of the *CHOP* mRNA, with the potential mir-615-3p binding site is depicted. For each binding site, the sequence alignment is shown as well as a schematic line diagram of the predicted complementary regions and bulges. The predicted minimum free energy (mfe) is depicted. Preferences were set to allow G:U wobble bases within the alignment; these are indicated in the figure.

*Due to the known proapoptotic role of CHOP under ER stress conditions, we next focused on miRs which might regulate CHOP expression and thus ER stress-induced apoptosis. Using computational tools we assessed each of the five miR's presented in Table 1 as potential regulators of CHOP, and we consequently narrowed our search to miR-615-3p as a miR which might regulate CHOP expression, which is encoded by the DNA-damage-inducible transcript 3 (Ddit3) gene. Computational searches yielded no fully conserved sites for miR-615-3p binding in the Chop 3′UTR across several species; however, several computational approaches predicted a single binding site for miR-615-3p in the mouse Chop 3′UTR (TargetScan, miRanda, miRWalk) (Figure 1A). We next identified a potential miR-615-3p binding site in the human CHOP 3′UTR based on minimum free energy (mfe) using the program RNAhybrid at* *http://bibiserv.techfak.uni-bielefeld.de/rnahybrid/* *[31]. Preferences were set to allow G:U wobble bases within the alignment; these are indicated in the figure 1B. For each binding site, the sequence alignment is shown as well as a schematic line diagram of the predicted complementary regions and bulges. While the exact binding sequence was not conserved, the relative position in the 3′UTR is the same. For reference, the last 6 bases shown of the miR-615-3p binding site on human CHOP (UUGGAG) correspond to the first 7 bases of the miR-615-3p binding site on mouse Chop (CUGAGGG) in a region of ∼68% identity by ClustalW alignment. Thus, we concluded that potential miR-615-3p binding sites exist in the 3′UTR of the CHOP gene in both mouse and humans.*

**Figure 2:**
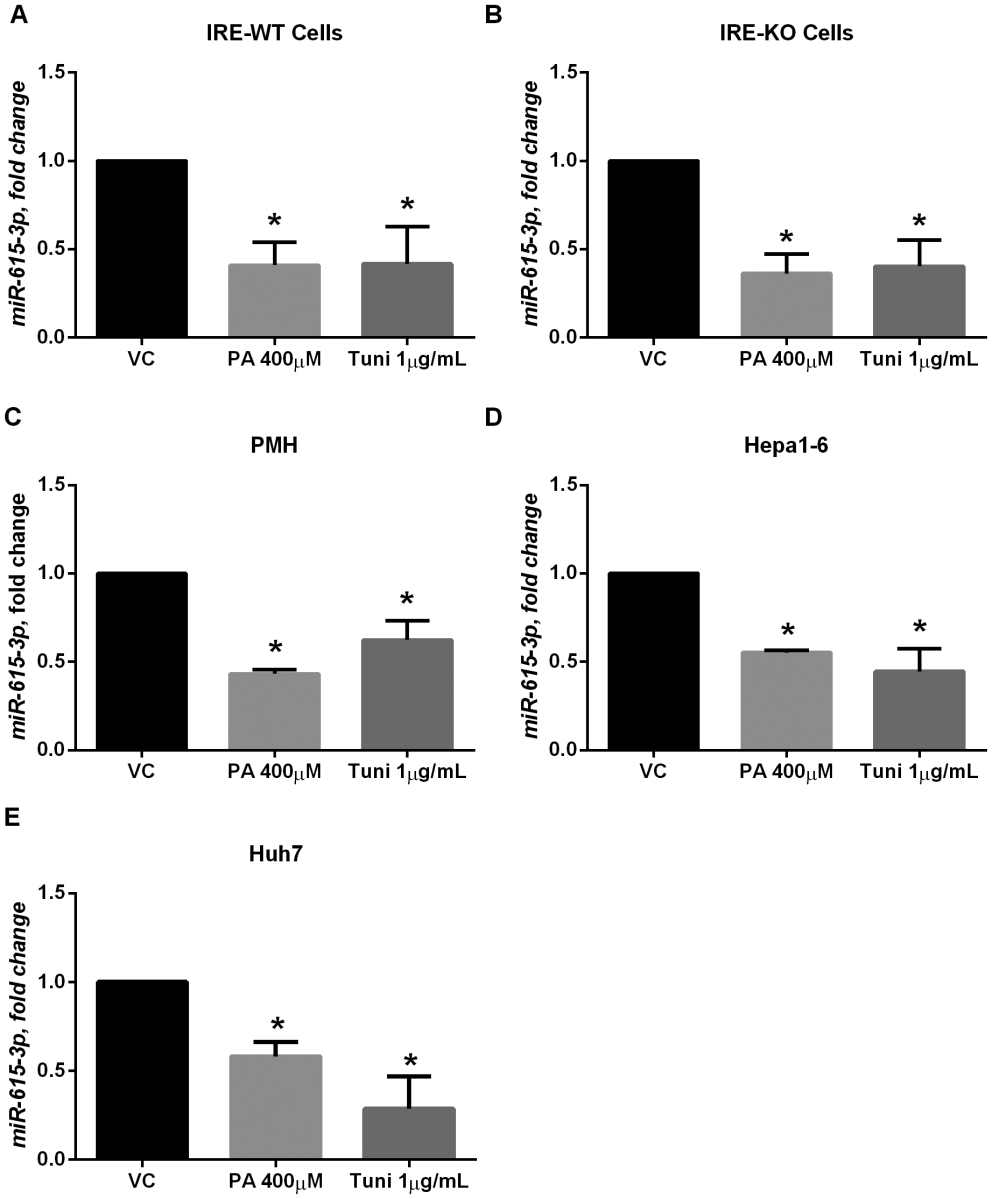
Palmitate treatment decreases miR-615-3p levels. (A) Neonatal IRE1α wild-type hepatocyte cells (IRE-WT) were treated with 400 µM palmitate (PA) or 1 µg/mL tunicamycin (tuni) for 16 hours. Control cells were treated with vehicle alone (VC). * P<0.05 compared to VC. (B) Neonatal IRE1α knockout (IRE-KO) cells were treated with 400 µM palmitate (PA) or 1 µg/mL tunicamycin for 16 hours. Control cells were treated with vehicle alone (VC). * P<0.05 compared to VC (C) Primary mouse hepatocytes (PMH) were treated with 400 µM palmitate (PA) or 1 µg/mL tunicamycin for 16 hours. Control cells were treated with vehicle alone (VC). * P<0.05 compared to VC. (D) Hepa1-6 cells were treated with 400 µM palmitate (PA) or 1 µg/mL tunicamycin for 24 hours. Control cells were treated with vehicle alone (VC). * P<0.05 compared to VC. (E) Huh7 cells were treated with 400 µM palmitate (PA) or 1 µg/mL tunicamycin for 30 hours. Control cells were treated with vehicle alone (VC). * P<0.05 compared to VC.

*We next confirmed our RNA sequencing data, that palmitate and tunicamycin treatment decreased miR-615-3p expression levels in IRE1α wild-type and knockout cells. We did not see a significant reduction in miR-615-3p following 12 hours of treatment with PA or tunicamycin (Figure S2A and S2B). A significant reduction in miR-615-3p levels was observed at 16 hours of treatment (Figure 2A and 2B). This occurred before significant apoptosis was observed in these cell lines (Figure S2C and S2D) and coincided with robust induction of CHOP expression (Figure S2E and S2F). CHOP protein levels were undetectably low in IRE-WT cells at 8 hours, and were detected at a low level at 8 hours in IRE-KO cells; however, the CHOP protein expression peaked at 16 hours, before the onset of apoptosis. Next, we verified that this occurred in isolated primary mouse hepatocytes and Hepa1-6 cells (Figure 2C and 2D). Indeed, in all four mouse cultured cells tested, palmitate and tunicamycin treatment significantly reduced miR-615-3p levels. Consistent with the mouse data, we observed a significant reduction in miR-615-3p in the human liver cell line, Huh7 (Figure 2E).*

### Mmu-miR-615-3p regulates *CHOP* expression

**Figure 3:**
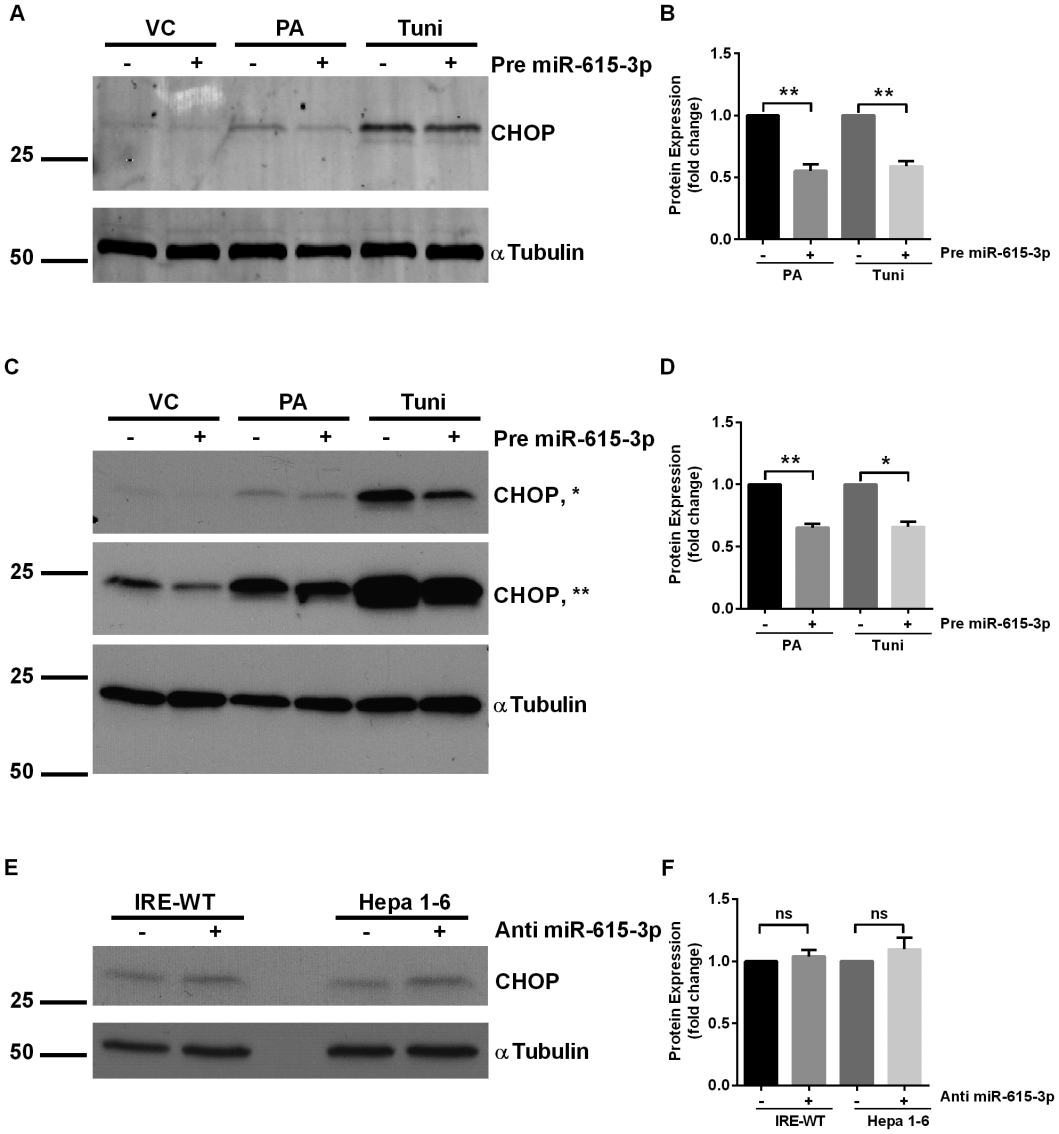
MiR-615-3p inhibits CHOP expression. (A) Representative western blot for CHOP in IRE-WT cells treated with 400 µM palmitate (PA) or 1 µg/mL tunicamycin (tuni) for 16 hours. The cells were transfected with either a negative control, or a precursor of miR-615-3p (pre-miR-615-3p). Molecular weights are indicated in kDa. The immune complexes were detected using an infrared fluorescent imaging system. Alpha-tubulin was used as loading control. (B) Quantification of CHOP protein levels normalized to tubulin, under the same conditions as A, expressed relative to the negative control mimic treated cells for each condition, respectively. ** P<0.01. (C) Representative western blot for CHOP in Hepa 1–6 cells treated with 400 µM palmitate (PA) or 1 µg/mL tunicamycin for 24 hours. The cells were transfected with either a negative control, or a precursor of miR-615-3p (pre-miR-615-3p). Molecular weights are indicated in kDa. The immune complexes were detected using enhanced chemiluminescence. Alpha-tubulin was used as loading control. The middle panel (**) depicts a longer film exposure of the top panel (*) (D) Quantification of CHOP protein levels normalized to tubulin, under the same conditions as C, expressed relative to the negative control mimic treated cells for each condition, respectively. * P<0.05, ** P<0.01 (E) Representative western blot for CHOP in IRE-WT and Hepa1-6 cells in cells transfected with either an antagomir to miR-615-p or a negative control antagomir. Alpha-tubulin was used as loading control. (F) Quantification of CHOP protein levels normalized to tubulin, under the same conditions as E, expressed relative to the negative control antagomir treated cells for each condition, respectively. P = ns.

*We next pursued a gain-of-function approach utilizing a mmu-miR-615-3p mimic to inhibit CHOP expression. As previously described by us and others [10], [32], low-level basal CHOP expression was detected in wild-type mouse liver cells (IRE-WT) and mouse hepatoma cell line, Hepa 1-6, Figure 3A and 3B, respectively. CHOP was induced by both PA and tunicamycin in these cells. Kinetic analysis of CHOP protein expression showed a significant increase in protein levels before the onset of apoptosis (Figures S2C-S2F). As predicted, in cells transfected with a precursor of miR-615-3p, PA-induced and tunicamycin-induced CHOP expression was significantly abrogated (Figure 3A – 3D). CHOP is induced transcriptionally under ER stress conditions. These data suggest that under ER stress conditions, once CHOP expression is induced, microRNA miR-615-3p confers a level of post-transcriptional regulation, such that the reduction of miR-615-3p under ER stress conditions, derepresses CHOP protein expression.*

*We next utilized a loss-of-function approach, using an antagomir targeting miR-615-3p. CHOP levels were low basally, and did not significantly increase with the antagomir treatment (Figure 3E), suggesting that miR-615-3p does not regulate basal levels of CHOP expression. These data are consistent with the concept that ER stress is required to increase CHOP expression [15]. Nor did we see a significant further increase in CHOP protein expression in PA- and tunicamycin-treated cells (Figure S3), suggesting that maximal CHOP expression under these conditions had already occurred due to the transcriptional upregulation of CHOP under ER stress conditions. Thus, as maximal expression was achieved, it could not be further increased by antagonizing mir-615-3p, levels of which are already reduced by both PA and tunicamycin treatment, as shown in Figure 2. Thus, under ER stress conditions, when CHOP expression is transcriptionally induced, miR-615-3p regulates its expression; by coordinately decreasing miR-615-3p levels maximal expression of CHOP is achieved by PA and tunicamycin.*

### Mmu-miR-615-3p binds the 3′UTR of *Chop*

**Figure 4:**
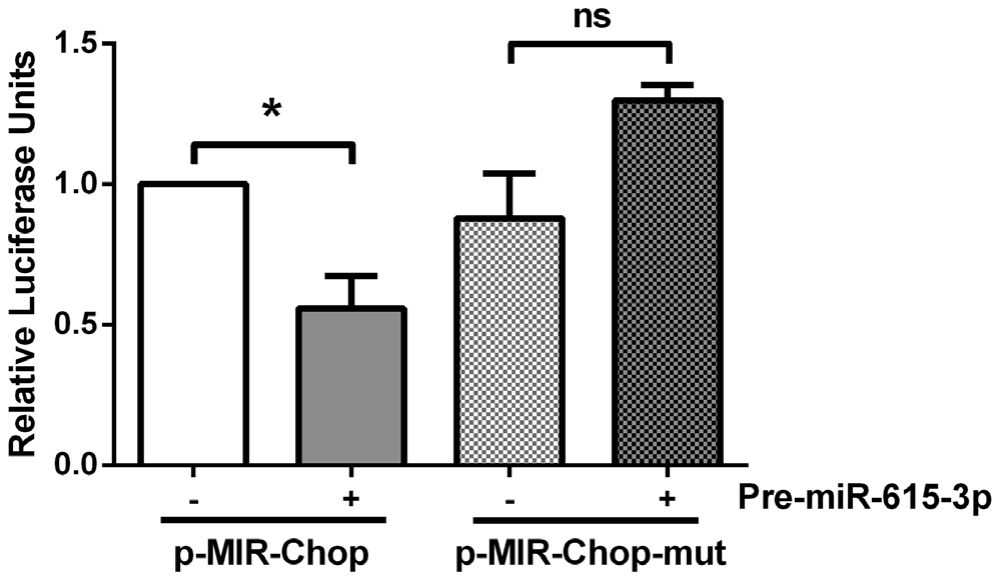
*Chop* mRNA is a direct target of miR-615-3p. A region of the *Chop* 3′UTR containing the putative miR-615-3p binding site was cloned into the pMIR-report vector downstream of the luciferase coding region (p-MIR-Chop). The 3′UTR segment with the putative miR-615-3p binding site mutated (p-MIR-Chop-mut) was also cloned into the pMIR-report vector. HEK293 cells were co-transfected with the respective reporter plasmid and precursor of miR-615-3p (pre-miR-615-3p) or a negative control precursor molecule. Relative luciferase activity (normalized to renilla) was measured 24 hours after the transfection. Data are expressed relative to the wild-type binding site transfected cells treated with a negative control precursor molecule and (n = 5 independent experiments), * P<0.05.

*We confirmed direct binding of the Chop mRNA by miR-615-3p by employing a luciferase-based reporter assay. The sequence of the miR-615-3p binding site in the Chop 3′UTR is shown in Figure 1A. This was cloned into the 3′UTR of the luciferase reporter pMIR-report vector as described under materials and methods. As a control, we mutated the seed sequence and strong complementarity along the full length of the binding site in the 3′UTR of the Chop transcript so as to abrogate miR-615-3p binding (Figure S1). In cells co-transfected with the reporter construct and miR-615-3p mimic, a significant reduction in the luciferase activity was observed (Figure 4). Furthermore, cells transfected with the mutated binding site containing reporter and the miR-615-3p mimic demonstrated no reduction in luciferase activity. These data confirm that miR-615-3p functionally and directly binds to the Chop 3′UTR.*

### MiR-615-3p mimic confers resistance to cell death

**Figure 5:**
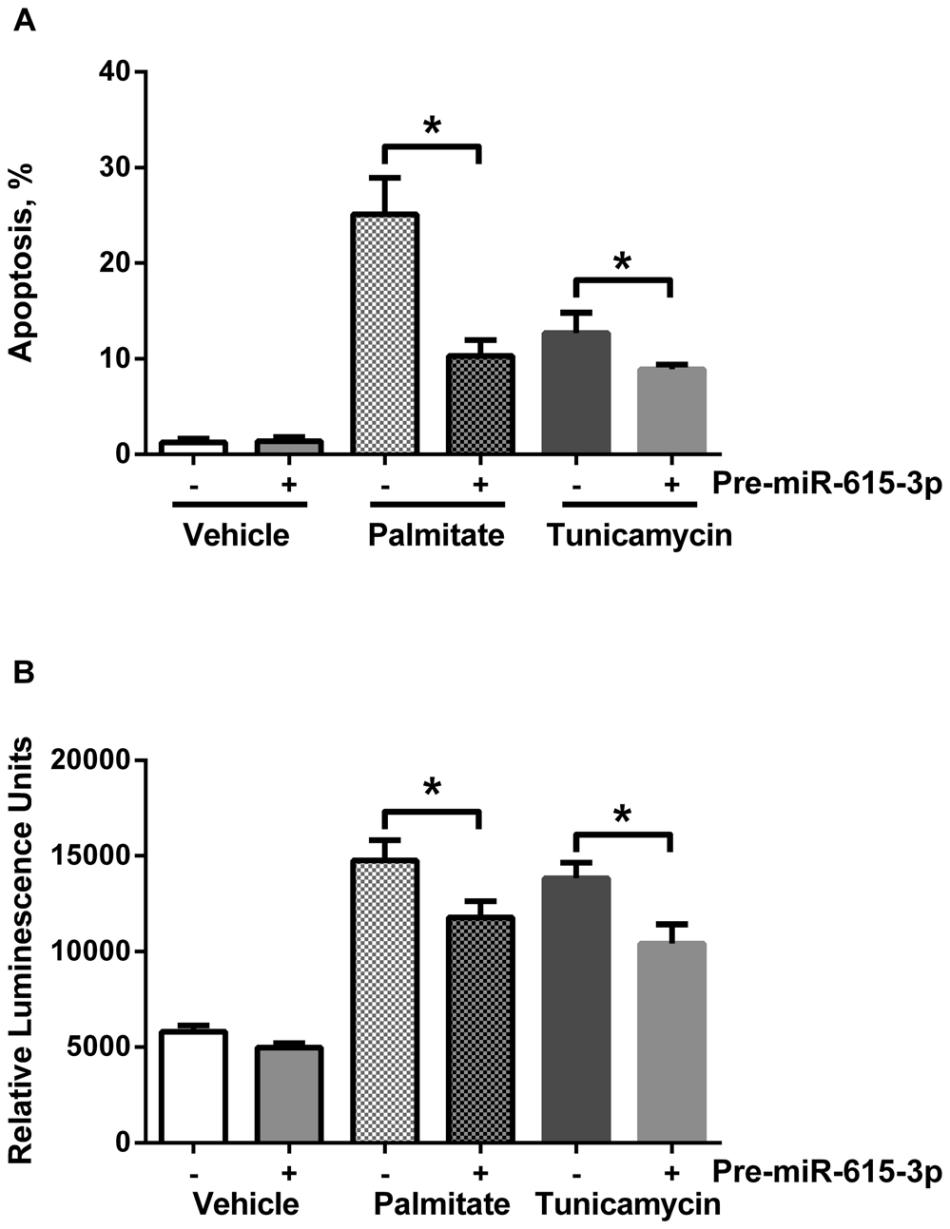
MiR-615-3p reduces palmitate-induced cell death. (A) Neonatal liver wild-type cells were transfected with either the negative control precursor or precursor of miR-615-3p, both fluorescently labeled with Cy3. 8 hours after transfection, cells were treated with 400 µM palmitate (PA) or 1 µg/mL tunicamycin for 18 hours. DAPI stained nuclei were counted in each condition. * P<0.05. (B) Neonatal liver wild-type cells were transfected with either the negative control precursor or precursor of miR-615-3p. 8 hours after transfection, cells were treated with 400 µM palmitate (PA) or 1 µg/mL tunicamycin for 18 hours. Caspase 3/7 activity was measured as described. * P<0.05.

*Having demonstrated a functional binding site for miR-615-3p in the Chop transcript, and regulation of CHOP protein levels by this mechanism, we next asked if miR-615-3p regulates sensitivity to PA-induced apoptosis. Utilizing a precursor of miR-615-3p, to inhibit CHOP expression, we observed a significant reduction in PA-induced and tunicamycin-induced apoptosis (Figure 5A). We confirmed apoptosis biochemically by caspase 3/7 activation (Figure 5B), which paralleled the morphologic assessment of apoptosis. As we utilized a fluorescently labeled precursor molecule, in our morphologic assessment, we only counted cells that were positively transfected. Our transfection efficiency was greater than 90%; however, in the caspase 3/7 assay all cells are assayed, therefore, the magnitude of reduction was somewhat mitigated. Furthermore, the miR-615-3p precursor only partially suppressed CHOP expression as shown in Figure 3, which corresponds with the partial resistance to apoptosis under similar conditions.*

### MiR-615-3p levels are reduced in nonalcoholic steatohepatitis

**Figure 6:**
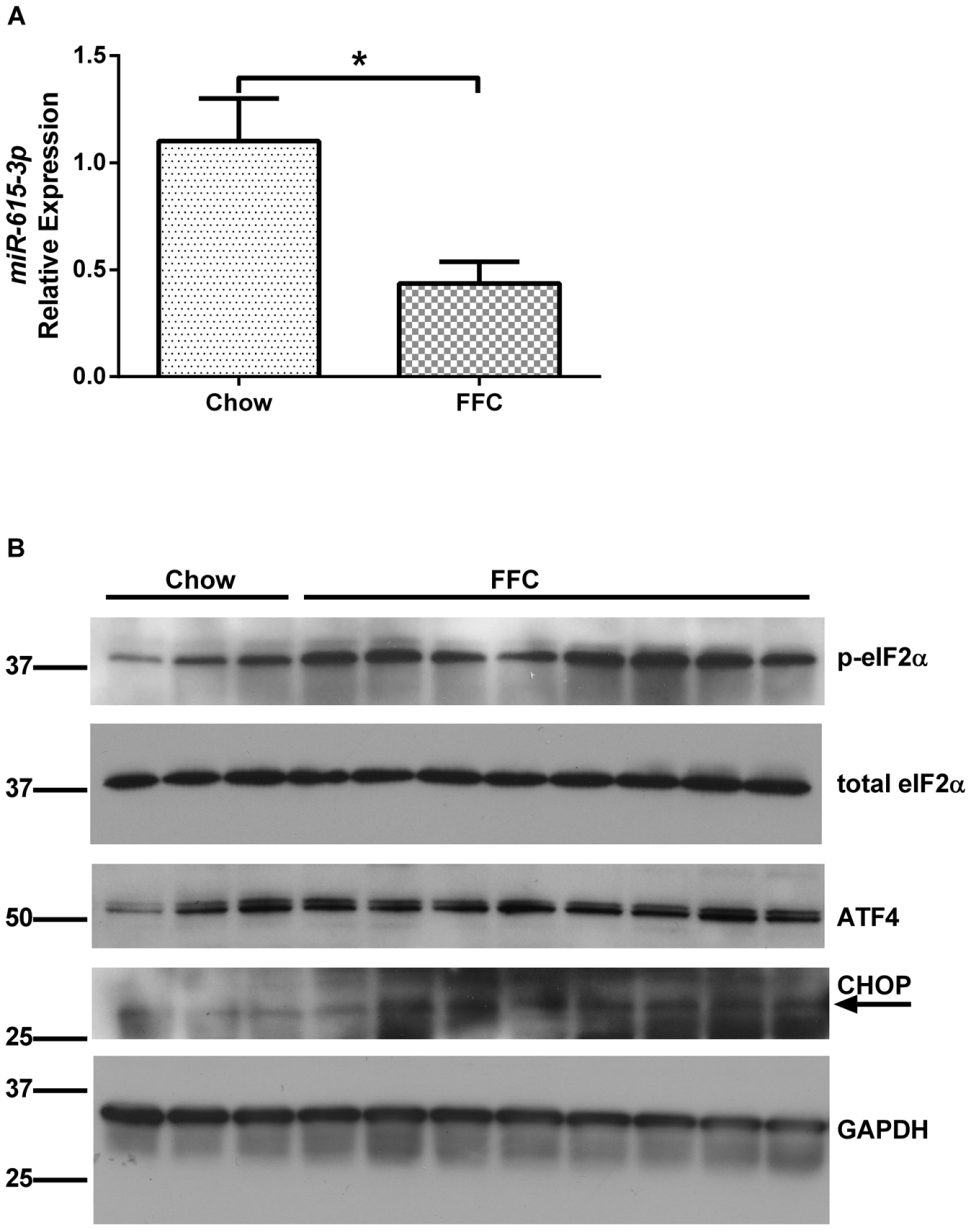
MiR-615-3p and CHOP levels in nonalcoholic steatohepatitis. (A) MiR-615-3p levels were measured in mouse liver from chow fed mice (n = 7) and mice with nonalcoholic steatohepatitis induced by feeding a diet high in fructose, fat and cholesterol (FFC) for 6 months (n = 13). Bars depict mean ±S.E.M, * *P*<0.05. (B) Immunoblots for p-eIF2α, total-eIF2 α, ATF4, CHOP and GAPDH for loading control from whole liver protein extracts from chow fed mice (n = 3) and FFC-fed mice (n = ). The arrow points to the predicted CHOP band in the immunoblot.

*To further explore the potential role for miR-615-3p in regulating lipoapoptosis in vivo, we interrogated the expression of this microRNA in archived liver RNA samples from chow fed mice and mice fed a diet high in fructose, fat and cholesterol (FFC) for 6 months. The liver injury and metabolic syndrome induced by this diet recapitulates human obesity-associated NASH and has been well characterized [30]. We detected miR-615-3p in whole liver RNA in chow fed mice. Furthermore, in FFC-fed mice the expression of miR-615-3p was significantly reduced (Figure 6A), consistent with our in vitro observations. CHOP protein expression was increased in FFC-fed mice (Figure 6B), as was the expression of ATF4, and phosphorylation of eIF2α, supporting in vivo activation of the protein kinase RNA-like ER kinase (PERK) signaling axis in FFC-fed mice. CHOP levels were very low in chow fed mice as previously reported [14], and induced by FFC feeding. We conclude that hepatic miR-615-3p levels are reduced by a diet high in fructose, fat and cholesterol, which might be a mechanism for the increase in CHOP protein expression. FFC-fed wildtype mice demonstrate a concomitant increase in hepatocyte lipoapoptosis (Idrissova et. al., under review).*

## Discussion

In this study we have demonstrated that the microRNA mmu-miR-615-3p is reduced under conditions of endoplasmic reticulum (ER) stress, wherein it regulates the expression of C/EBP homologous protein (CHOP) and determines cellular sensitivity to cell death. The principal findings that support these conclusions are: 1) mmu-miR-615-3p levels are reduced under conditions of ER stress; 2) there is a functional miR-615-3p binding site in the 3′UTR of the *Chop* mRNA; and 3) ER stress-induced cell death is reduced by miR-615-3p-dependent CHOP repression.

In the current study we utilized mouse hepatocyte cells lines, primary mouse hepatocytes and a human hepatocellular carcinoma-derived cell line. MicroRNA miR-615-3p was detected in five different hepatocyte cell lines. We demonstrate that under palmitate-induced ER stress the levels of miR-615-3p are reduced in all five cell lines tested. It is also detected in rat cholangiocyte cell lines (Dr. LaRusso, personal communication). We used palmitate to induce ER stress, as it is a physiologically abundant free fatty acid, whose levels are further elevated in obesity-associated fatty liver disease, wherein, it is implicated as the stimulus for cellular demise[4], [5]. We additionally chose to include tunicamycin as an independent inducer of ER stress as a positive control [33]. We and others have shown that palmitate treatment activates the proximal unfolded protein response sensors and leads to CHOP expression [11], [12], [13], [32]. These studies have mechanistically demonstrated an increase in membrane lipid saturation, and ER calcium depletion upon palmitate loading.

Mmu-miR-615-3p has a single predicted binding site in the *Chop (ddit3)* 3′UTR. The *Chop* 3′UTR is one hundred and ninety five nucleotides in length, with few computationally identified microRNA binding sites. Additionally, we have identified a potential miR-615-3p binding site in the human *CHOP* 3′UTR. A microRNA which is transcriptionally regulated by CHOP, miR-708, has been previously described [34]. This microRNA is induced by ER stress, and represses rhodopsin, thus limiting the amount of new rhodopsin entry in to the ER. Mir-211 is transcriptionally induced under ER stress conditions by PERK and ATF4, with subsequent repression of CHOP transcription by causing promoter hypermethylation [35]. ER stress repressed the cluster miR-106b∼25 via ATF4 and Nrf2 allowing increased Bim protein expression and apoptosis [36]. MiR-615-3p adds to the growing cache of microRNAs linked to the ER stress response. Augmentation of miR-615-3p activity by exogenous administration of a precursor repressed CHOP levels; however, we did not observe any further augmentation of the expression of CHOP by antagonizing miR-615-3p. Our data suggests that the reduction of this microRNA by palmitate facilitates maximal expression of CHOP protein under conditions of palmitate-induced ER stress, but does not contribute to its basal regulation. Furthermore, we observed a similar reduction in miR-615-3p levels in a mouse liver cell line deficient in IRE1α. This suggests that miR-615-3p is not targeted by the RNase activity of IRE1α. However, this does not exclude regulation of miR-615-3p by the other signaling pathways that comprise the UPR, viz. PERK and ATF6. Systematic dissection of these pathways will be our focus in future studies.

Palmitate-induced hepatocyte lipoapoptosis is thought to be one of the key cellular mechanisms underlying disease onset and progression in nonalcoholic steatohepatitis. Palmitate induces ER stress in hepatocytes and many other cell types. We have now demonstrated that palmitate-induced apoptosis is partially CHOP-dependent. The miR-615-3p precursor partially reduced palmitate and tunicamycin-induced CHOP levels. This is likely why a partial reduction in palmitate and tunicamycin-induced apoptosis was observed. A previous study had demonstrated that the genetic deletion of CHOP did not protect the hepatocyte from palmitate-induced apoptosis [32]. However, in this study hepatocytes were treated with 200 µM palmitate for 16 hours; these conditions in our hands do not result in significant apoptosis [23], [24]. In the present study a higher concentration of palmitate was used, i.e., 400 µM, and this resulted in significant apoptosis at a longer duration of treatment (24 hours). Furthermore, others have demonstrated a reduction in palmitate-induced apoptosis upon silencing of CHOP expression in human hepatoma cells [10]. In these studies we have not focused on events downstream of CHOP induction; however, recent literature suggests a possible induction of the TRAIL receptor, death receptor 5 by CHOP as a pro-apoptotic signal under ER stress conditions [17]. Alternatively, increased protein synthesis has been implicated as a mechanism for CHOP-induced cell death [16]. In the later study DR5 was not a transcriptional target of CHOP. Therefore, it will be interesting to observe in future studies which of these mechanisms is operational during hepatocyte lipoapoptosis.

In this study we have demonstrated a reduction in miR-615-3p by palmitate. Palmitate can repress gene expression by epigenetic changes resulting in promoter hypermethylation [37]. As the miR-615-3p coding region is located within intron 1 of the *Hoxc5* gene on chromosome 15, these data suggest that palmitate can repress the expression of this genetic locus. This is one possible explanation for palmitate-induced reduction in miR-615-3p levels, and will require further studies to elucidate, which is not the focus of the current manuscript. We do provide evidence that repression is independent of the ER-stress responsive kinase and RNase IRE1α, as we observed similar repression in IRE1α-deficient cells.

MicroRNAs are altered in the liver in NASH, and it has been proposed that circulating microRNAs or microRNAs present on microvesicles may serve as a biomarker for NASH. Our observations have identified a new candidate microRNA, miR-615-3p, levels of which are significantly repressed in the liver in NASH for such studies.

In summary, these data support a model that palmitate lowers miR-615-3p levels, thus derepressing CHOP expression under conditions of ER stress, consequently promoting lipoapoptosis. We have identified a mechanism for ER stress-induced apoptosis which relies on the maximal expression of CHOP by the inducing stimulus, palmitate, in this case. We suggest that augmentation of miR-615-3p activity is an attractive target for decreasing lipoapoptosis.

## Supporting Information

Figure S1 **Sequences of wild-type and mutated miR-615-3p binding sites in** ***Chop*** **3′UTR.** (A) Sequence of the region containing the predicted binding site for miR-615-3p in the 3′UTR of the *Chop* transcript was cloned into the pMIR reporter to generate a luciferase reporter plasmid, we have designated pMIR-ddit3, as described in Materials and Methods. The nucleotides which were altered to generate a mutated plasmid, which we designated pMIR-ddit3-mut are underlined. The seed region complementary nucleotides are in bold. (B) The sequence of the mutated pMIR-ddit3-mut is depicted. The altered nucleotides are in italics. (C) Mutagenesis of the miR-615-3p binding site within the mouse *Chop* sequence was designed to eliminate the strong complementarity along the full length of the binding site. Introduced mismatches are indicated with an ‘x’.
(TIF)

Figure S2 **Kinetics of miR-615-3p decrease, CHOP expression and cell death.** (A) Sequence of the region containing the predicted binding site for miR-615-3p in the 3′UTR of the *Chop* transcript. (B) Apoptosis assessed by DAPI stained nuclear morphology following treatment with vehicle control (VC), 400 µM palmitate, or 1 µg/mL tunicamycin for 16 hours and 24 hours. Bars depict mean ±SEM, * p<0.05, compared to VC, 24 h.
(TIF)

Figure S3 **Antagonism of miR-615-3p does not increase CHOP expression.** Immunoblots for CHOP in (A) IRE-WT and (B) Hepa1-6 cells transfected with either an antagomir to miR-615-p or a negative control antagomir, and treated with vehicle control (VC), 400 µM palmitate, or 1 µg/mL tunicamycin for 16 hours. Alpha-tubulin was used as loading control.
(TIF)

Data S1 **MicroRNAs downregulated in IRE-WT (Vehicle control versus palmitate).**
(XLSX)

Data S2 **MicroRNAs downregulated in IRE-WT (Vehicle control versus tunicamycin).**
(XLSX)

Data S3 **MicroRNAs downregulated in IRE-KO (Vehicle control versus palmitate).**
(XLSX)

Data S4 **MicroRNAs downregulated in IRE-KO (Vehicle control versus tunicamycin).**
(XLSX)
