# Supplementary figures and images for: Mmu-miR-615-3p Regulates Lipoapoptosis by Inhibiting C/EBP Homologous Protein
Source: PLoS One. 2014 Oct 14;9(10):e109637. doi: 10.1371/journal.pone.0109637 (PMC4196923; doi:10.1371/journal.pone.0109637)

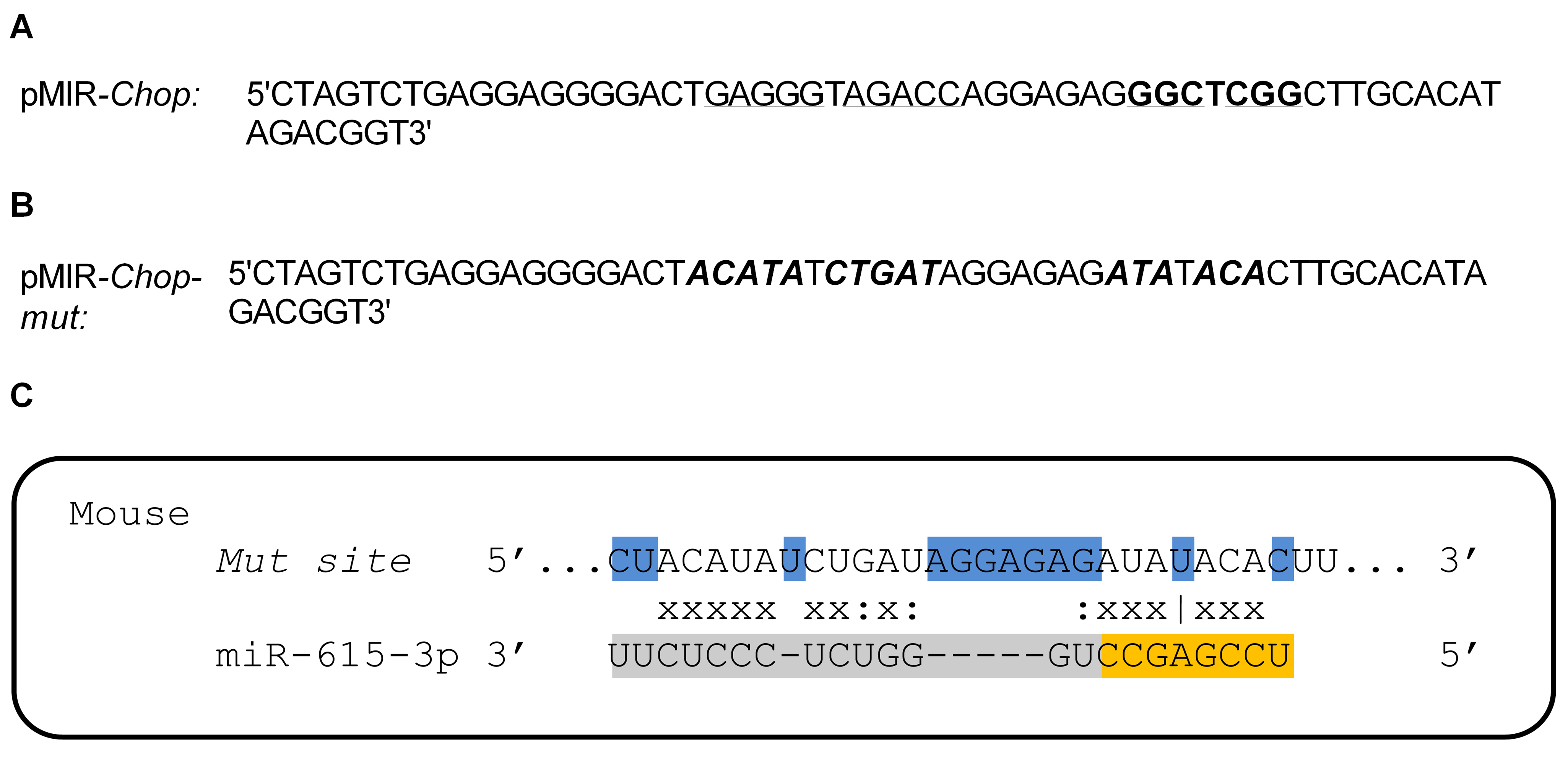

Supplement: Figure S1 — Sequences of wild-type and mutated miR-615-3p binding sites in Chop 3′UTR. (A) Sequence of the region containing the predicted binding site for miR-615-3p in the 3′UTR of the Chop transcript was cloned into the pMIR reporter to generate a luciferase reporter plasmid, we have designated pMIR-ddit3, as described in Materials and Methods. The nucleotides which were altered to generate a mutated plasmid, which we designated pMIR-ddit3-mut are underlined. The seed region complementary nucleotides are in bold. (B) The sequence of the mutated pMIR-ddit3-mut is depicted. The altered nucleotides are in italics. (C) Mutagenesis of the miR-615-3p binding site within the mouse Chop sequence was designed to eliminate the strong complementarity along the full length of the binding site. Introduced mismatches are indicated with an ‘x’. (TIF) [file pone.0109637.s001.tif]

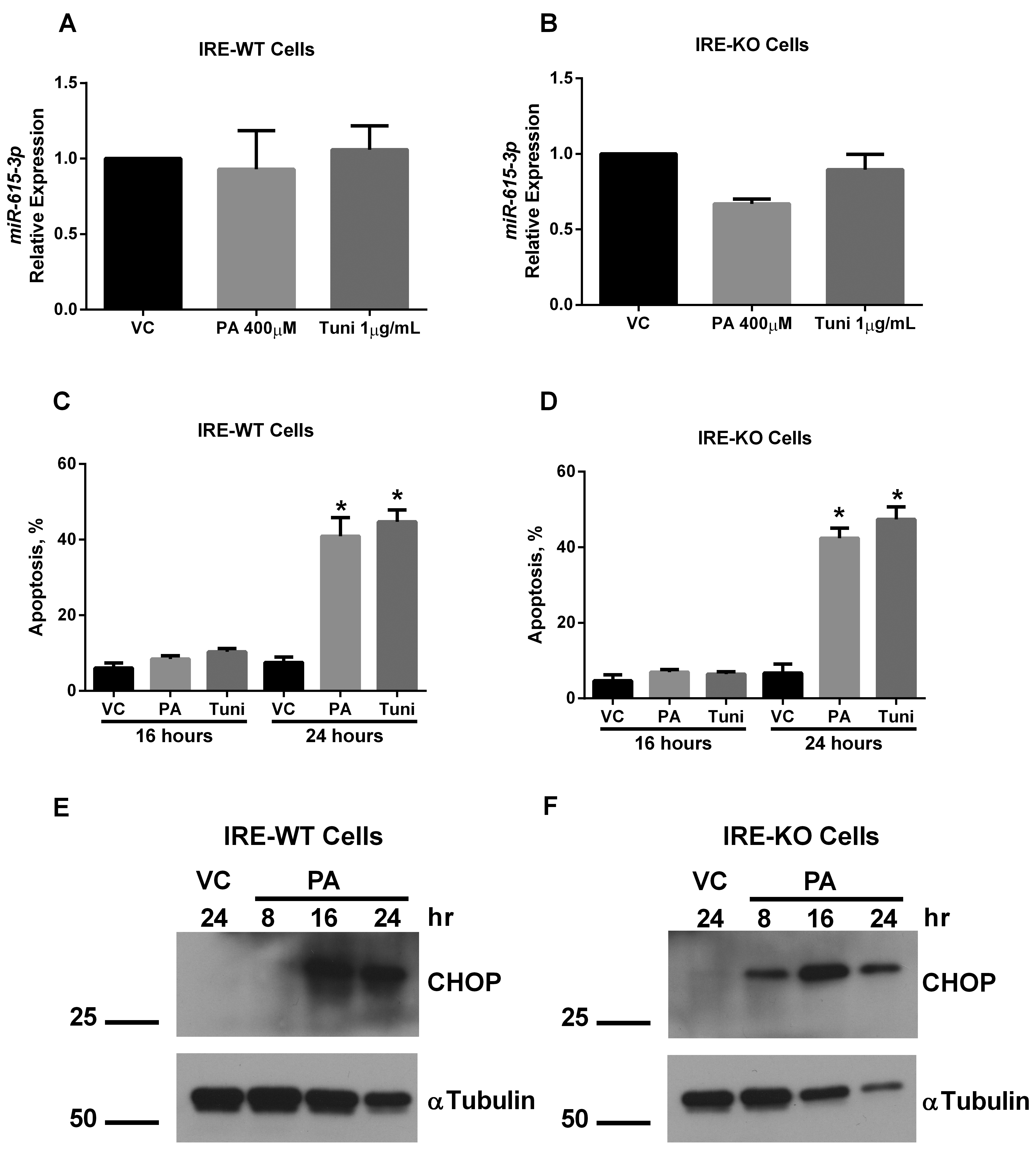

Supplement: Figure S2 — Kinetics of miR-615-3p decrease, CHOP expression and cell death. (A) Sequence of the region containing the predicted binding site for miR-615-3p in the 3′UTR of the Chop transcript. (B) Apoptosis assessed by DAPI stained nuclear morphology following treatment with vehicle control (VC), 400 µM palmitate, or 1 µg/mL tunicamycin for 16 hours and 24 hours. Bars depict mean ±SEM, * p<0.05, compared to VC, 24 h. (TIF) [file pone.0109637.s002.tif]

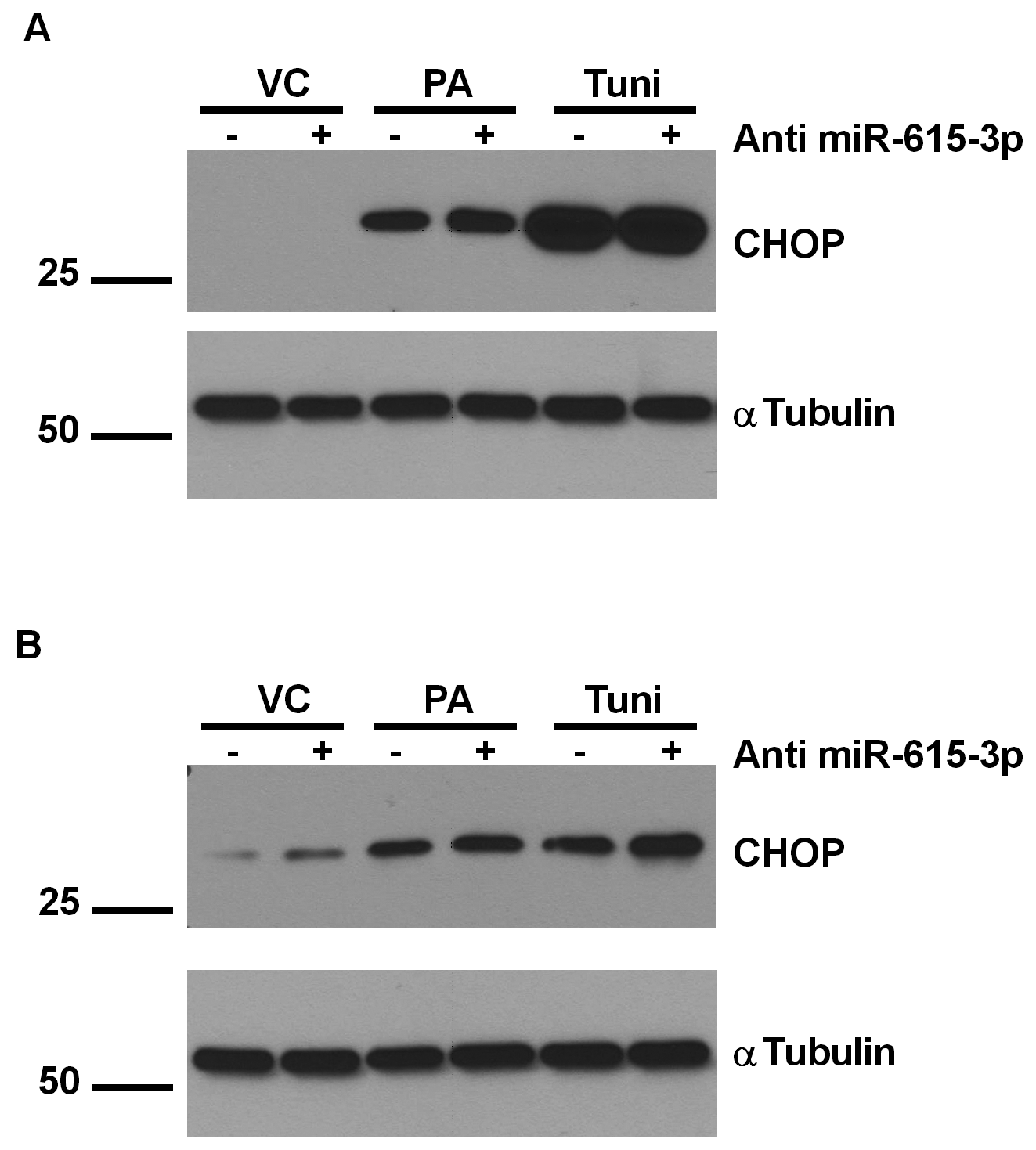

Supplement: Figure S3 — Antagonism of miR-615-3p does not increase CHOP expression. Immunoblots for CHOP in (A) IRE-WT and (B) Hepa1-6 cells transfected with either an antagomir to miR-615-p or a negative control antagomir, and treated with vehicle control (VC), 400 µM palmitate, or 1 µg/mL tunicamycin for 16 hours. Alpha-tubulin was used as loading control. (TIF) [file pone.0109637.s003.tif]
